# Long-Term Patency of Venous Conduits Targeting the Right Coronary Artery System—Single Is Superior to Sequential bypass Grafting

**DOI:** 10.3390/jcdd9090285

**Published:** 2022-08-26

**Authors:** Rawa Arif, Aglaia Warninck, Mina Farag, Wiebke Sommer, Florian Leuschner, Norbert Frey, Matthias Karck, Gregor Warnecke, Nicolas A. Geis

**Affiliations:** 1 Department of Cardiac Surgery, University Hospital Heidelberg, 69120 Heidelberg, Germany; 2 Department of Cardiology, Angiology and Pneumology, University Hospital Heidelberg, 69120 Heidelberg, Germany

**Keywords:** coronary artery disease (CAD), coronary artery bypass grafting (CABG), coronary artery graft patency, right coronary artery

## Abstract

Objective: Little is known about the fate of bypass grafts to the right coronary system. To investigate the long-term patency of venous bypass grafts directed to the right coronary artery (RCA) based on postoperative angiograms and to identify predictors of graft occlusion. Methods: In this single-center study, all patients who underwent coronary angiography from 2005 to 2021 after previously undergoing isolated coronary artery bypass grafting (CABG) were included. The primary endpoint was graft occlusion over a median follow-up of 9.1 years. Results: Among a total of 1106 patients (17.0% women, 64 (57–71) years median age), 289 (26.1%) received a sequential vein graft and 798 (72.2%) a single graft. Multivariate regression revealed age (HR 1.019, CI 95% 1.007–1.032), the urgency of CABG (HR 1.355, CI 95% 1.108–1.656), and severely impaired left ventricular function (HR 1.883, CI 95% 1.290–2.748), but not gender and chronic total occlusion (CTO) as predictive factors for graft occlusion. Single conduits were found to be a predictor of graft patency (HR 0.575 CI 95% 0.449–0.737). The angiographic outcome showed an overall 10-year freedom from graft occlusion of 73.4% ± 1.6%. The 5-year (10-year) freedom from graft occlusion was 76.9% ± 2.8% (57.8% ± 4.0%) for sequential grafts and 90.4% ± 1.1% (77.8% ± 1.7%) for single grafts (log-rank *p* < 0.001). Conclusions: In symptomatic patients with renewed angiography, venous bypass grafting of the RCA showed acceptable long-term patency rates. Single bypass grafting of the RCA was superior to sequential grafting, which needs to be further investigated.

## 1. Introduction

Coronary artery bypass grafting (CABG) remains a standard procedure for revascularization of three-vessel disease/coronary artery disease (CAD) [1,2]. Despite experiences of over half a century, the conduit of choice remains a matter of debate in terms of long-term patency, further sustained by the lack of evidence due to inherent difficulties in conducting prospective studies. Moreover, interobserver bias and the heterogeneity of patients further contribute to this dilemma. The general opinion that arterial grafts are superior to venous grafts has been demonstrated for revascularization of the left coronary artery (LCA), defining the synopsis of the current guidelines [3,4,5,6]. This phenomenon may originate from several factors (1) smaller vessel diameters of the LCA compared to the right coronary artery system; (2) mainly diastolic perfusion within the left system with consecutively less competitive blood flow as opposed to diastolic/ systolic blood flow in the RCA; as well as (3) reduced wall stress due to smaller muscle mass of the right ventricle. Venous graft disease has been the topic of several clinical studies and ongoing basic research [7,8]. Within six weeks, the development of intimal hyperplasia and arteriosclerosis in the venous grafts lead to graft occlusion in approximately 50% of conduits over ten years, with some investigations reporting even worse patency rates [9,10,11]. Whether the storage solutions of the venous conduits or the preimplantation manual dilation are responsible for endothelial damage, activation of the endothelium, and inflammation, has not been distinguished yet.

Despite standard recommendations preferring arterial revascularization, even the largest randomized controlled trial has not been able to show benefits in terms of survival when bilateral internal thoracic artery (ITA) CABG was performed instead of single ITA [4]. In addition, in this trial, ITA grafts to the RCA were prohibited due to concerns about long-term patency. Moreover, the fate of the RCA system has not been addressed in subpopulation analysis or other reports. Thus, the optimal revascularization strategy for the RCA remains a matter of ongoing investigation.

This study aims to analyze the long-term patency of venous conduits utilized for the revascularization of the right coronary artery and determine possible risk factors associated with graft occlusion. 

## 2. Patients and Methods

### 2.1. Patients

**Figure 1:**
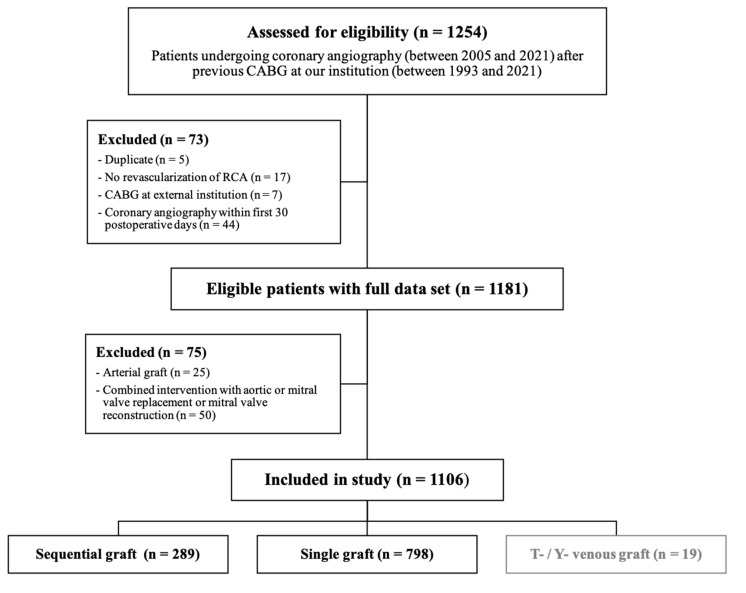
CONSORT diagram.

The cohort comprises a total of 1106 patients. All patients underwent isolated CABG (without concomitant procedures such as a valve or aortic interventions) with revascularization of the RCA utilizing a saphenous venous graft (SVG) at our institution. However, “sequential” RCA grafts also included bypasses coming via the left coronary system. Angiographic investigations were performed between 2005 and 2020. All patients underwent CABG between January 1993 and January 2021. During this period, 16,194 isolated CABG procedures were performed at our institution. Of those, 7898 patients received an RCA bypass graft. We excluded all patients who underwent coronary angiography within the first 30 postoperative days (44 patients (including three arterial bypass grafts). Graft occlusion was detected in fifteen patients (including one arterial bypass graft) and five patients deceased within 30 days) since the need for early cardiac catheterization is usually due to periprocedural adverse events, which does not reflect the objective of our investigation. Angiographic investigations for patients with a history of CABG performed at an external institution were also excluded. The study design is depicted in the CONSORT diagram (Figure 1).

Due to the retrospective nature of the study, the need for informed consent was waived by the local Research Ethics Committee of the University of Heidelberg, and the study was subsequently approved (S-942/2020, 14 December 2020).

### 2.2. Data Collection

Data sets were collected from medical records consisting of over 5000 variables for each patient. Included were demographic characteristics, cardiovascular risk profile, diagnostic methods, patients’ preoperative clinical status, details of the surgery, postoperative outcome, and angiography findings. The maximum of institutional follow-up was included. All available preoperative and postoperative angiographies and their reports have been analyzed and verified by experienced interventional cardiologists and cardiac surgeons. If multiple angiographies were detected for a patient, the earliest described graft occlusion was documented in this study. Graft occlusion was defined as the complete cessation of blood flow in the respective vessel. Furthermore, later interventions concerning the native coronary arteries (CA) or grafts were also included. 

### 2.3. Statistical Analysis

Continuous variables were expressed as mean ± standard deviation or median and interquartile range (IQR) in data not normally distributed, and categorical variables as absolute and relative frequencies. Student’s *t*-test and Pearson’s χ^2^ were used for continuous and categorical variables, respectively. 

To investigate the influence of different patient characteristics and procedural factors associated with graft occlusion, a cox hazard univariate logistic mixed effect model was performed, containing factors that have been proven to influence outcomes after CABG. Aiming to investigate the influence of additional factors on graft patency, we analyzed parameters such as measured graft blood flow, coronary diameter, and the number of sequential anastomses, utilizing a cox hazard logistic mixed effect regression, after dividing the cohort into a single graft and sequential graft subgroups. The influence of blood flow was not analyzed for the total cohort since flow rates of sequential grafts are not comparable to flow rates of single anastomosis grafts if not measured within the respective sequence. Nineteen patients who received a T-/Y-venous graft were included in the overall patency analysis but not further analyzed due to sample size limitations. Due to the exploratory character of this analysis, all *p* values are of descriptive nature. A *p* value < 0.05 was considered significant. All analyses were performed using SPPS Statistics 28 (IBM Corp., Armonk, NY, USA) and GraphPad Prism 9.3.1 (GraphPad Software, San Diego, CA, USA).

## 3. Results

### 3.1. Demographics and Clinical Presentation

A total of 1106 patients were included in the study after exclusion according to the above-mentioned criteria. The median patient age was 64 (IQR 57–71) years at the time of initial CABG, of whom 188 (17%) were female, with a median logistic EuroScore of 6.05% (IQR 3.14–10.43). Thirty-five percent of the patients underwent previous percutaneous coronary intervention (PCI), and 3.5% had previous cardiac surgery. Cardiovascular risk factors were distributed as shown in Table 1, wherein 50% of patients presented with a history of smoking. Atrial fibrillation and severely impaired LV function were detected in 7.6% and 10.8% of the patients, respectively. Further demographic details are depicted in Table 1. 

A mean number of 2.81 ± 0.65 grafts were utilized for revascularization. From 1106 patients, 289 (26.1%) received sequential vein grafting, 798 (72.2%) single vein- and 19 (1.7%) a T-/Y-venous graft. Chronic total occlusion (CTO) of the RCA was preoperatively detected in 174 (15.7%) patients. Visible collateralization of the RCA with distinguishable targeting vessels (ramus marginals dexter [RMD]; right posterior descending [RPD] or ramus posterolateralis dexter [RPLD]) was observed in 95 of 174 (55%) CTOs in preoperative angiography.

### 3.2. Patency Rate of the Venous Bypass Conduit

The median period between CABG and the latest angiography was 9.1 (IQR 4.5–14.3) years. The overall graft occlusion rate was 33.3%. The native RCA intervened in 15.8%, and the SVG directed to the RCA intervened in 11.8% of the cases during follow-up. The coronary angiography findings, as well as graft characteristics, can be found in Table 2. Intraoperative findings, graft localizations, postoperative complications, time to angiography stratification, and indication for angiography are shown in supplement tables (Supplementary Tables S1–S4). 

**Figure 2:**
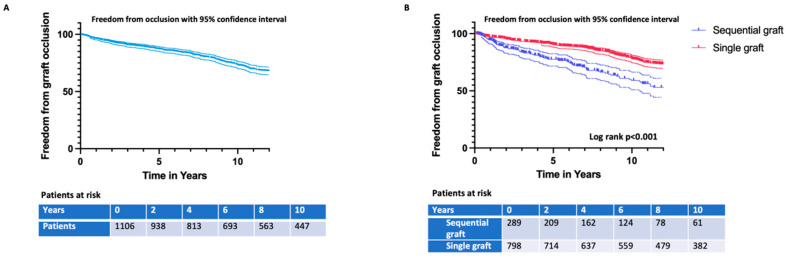
(**A**): Overall freedom from graft occlusion. (**B**): Freedom from graft occlusion for different graft types: single conduit and sequential conduit.

The overall 1-, 5-, and 10-year freedom from graft occlusion rate was 96.5 ± 0.6%, 87.4 ± 1.1%, and 73.4 ± 1.6%, respectively (Figure 2A). The 1-, 5-, and 10-year freedom from graft occlusion rate was 94.2 ± 1.4%, 76.9 ± 2.8%, and 57.8 ± 4.0% for sequential grafts and 97.1 ± 0.6%, 90.4 ± 1.1%, and 77.8 ± 1.7% for single grafts (log-rank *p* < 0.001) (Figure 2B).

Of note, an additional analysis comparing bypass graft patency of single versus sequential conduits without censoring patients that underwent coronary angiography within the first 30 postoperative days (additional 3/12 single RCA bypass occlusions and 11/29 sequential bypass graft occlusions) also showed superior patency rates of single RCA bypass grafts in comparison to sequential grafts (*p* < 0.001, data not shown).

In addition, when comparing single and sequential bypass grafts, analysis of “graft failure” (defined as occlusion or severe stenosis ≥75%) also showed the superiority of single RCA bypass grafts (*p* < 0.001, data not shown). A combined endpoint analysis (graft occlusion, stenosis ≥75% and/or bypass intervention) also revealed lower event rates in patients with single bypass grafts (*p* < 0.001, data not shown).

Out of 289 sequential grafts in 70 (24.6%) patients, only the RCA segment was sequentially targeted with detected total graft occlusion in 28 (40.0%) patients during follow-up. In 215 (75.4%) patients, both the right and left coronary system was targeted 215 (75.4%). Thereof total occlusion was detected in 73 (34.0%) patients. Of 73 occluded sequential grafts targeting both RCA and LCA, 22 grafts were patent within the LCA system and 51 occluded on the level of the neo-ostium at the proximal aortic anastomosis (four missing values for both). 

### 3.3. Regression Analysis

Univariate regression analysis revealed several influencing factors on RCA conduit patency, which are listed in Table 3. The type of grafting technique was significantly correlated to patency since single grafts reduced the risk of occlusion almost by half in this cohort (HR 0.567 CI 95% 0.450–0.71, *p* < 0.001). CTO showed no influence on patency. Impaired LV function and atrial fibrillation have been detected as possible risk factors, as well as BMI. Age was found to be a significant risk factor for early graft occlusion (HR 1.024, CI 1.013–1.036, *p* < 0.001). The grade of RCA-stenosis did not correlate with graft patency in this investigation (*p* = 0.255).

After including significant influencing univariate factors in a mixed effect model, multivariate analysis revealed age, grafting technique, impaired LV function, and urgency of CABG procedure as significant risk factors for long-term graft occlusion (Table 3). 

Furthermore, within the subgroup of single bypass conduits, the lumen diameter of the target vessel (mean 1.578 ± 0.3 mm) did not show a relevant influence on graft occlusion (*p* = 0.56), whereas the measured blood flow (61.37 ± 34 mL/min) was detected as a risk factor for graft occlusion in univariate analysis (HR 0.992 CI 0.987–0.997; *p* = 0.002). To distinguish whether multiple sequential anastomoses affect graft occlusion, we performed a regression analysis for sequential grafts (2.06 ± 0.639 anastomoses, range 1–4), showing no significance for multiple anastomoses in a sequential graft (*p* = 0.335).

## 4. Discussion

**Figure 3:**
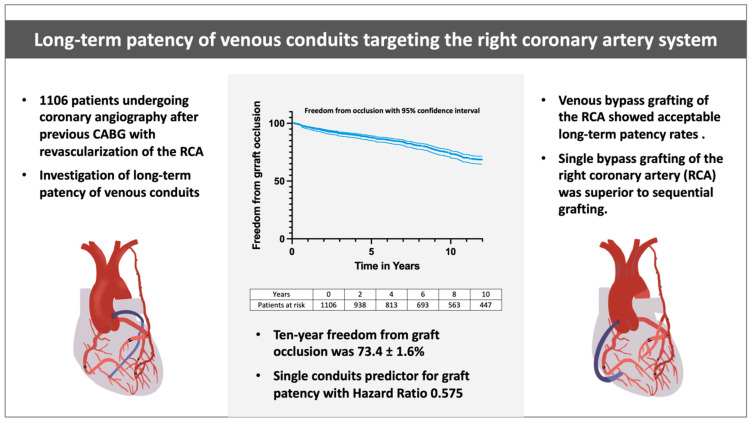
Overview of the study rationale, important findings, and conclusion. The overall freedom from graft occlusion is depicted as the central figure. Representative figures of mixed revascularization with a sequential venous conduit on the left and a single venous conduit to the right coronary artery on the right side.

We present a single-center retrospective cohort analysis, investigating long-term venous bypass graft patency to the right coronary system and determining risk factors associated with graft occlusion. Our analysis revealed a more than acceptable overall patency rate for saphenous vein grafts (SVG) targeting the RCA of 73 % after ten years in a cohort that has undergone repeated angiography, mainly for symptomatic reasons rather than for study purposes (Figure 3). 

The venous conduits target the RCA proof of acceptable long-term durability, despite controversial findings from previous studies [10,11]. However, Goldman et al. reported a 10-year patency rate of SVGs to the RCA of only 56% in a nowadays historic cohort [10]. In another study, Fitzgibbon et al. described a 45% occlusion rate after >6.5. years of vein grafts targeting the RCA [12]. Ruttmann et al. found in a subgroup analysis a lower risk for graft occlusion when the radial artery (RA) was grafted to the RCA compared to the usage of SVG. Furthermore, 99.2% of all SVGs were single anastomosis grafts, of which 43% showed occlusion after 6.5 years [11]. 

In this study, the grafting technique proved most influential, resulting in a significantly higher patency rate for single bypass conduits to the RCA compared to sequential vein-grafting. This finding is in keeping with an analysis from the PREVENT IV trial, demonstrating a higher one-year venous graft failure for sequential conduits compared to single SVGs [13]. On the other hand, Li et al. provided a systematic review reporting a risk reduction of 0.67 (95% CI 0.60–0.74) for occlusion of sequentially anastomosed venous grafts compared to single grafts [14]. The same study showed that side-to-side anastomoses of the sequential grafts were more patent than end-to-side anastomoses. However, the comparison between single grafts and end-to-side anastomoses showed comparable patency rates. Nevertheless, both studies did not distinguish targeted coronary systems. Several pathophysiological factors may be responsible for superior patency rates of single conduits to the RCA: 

First, the RCA—proximal to the crux cordis—usually provides larger vessel diameters compared to the typically targeted LCA branches. Consecutively, the RCA system is more susceptible to competitive blood flow than the LCA system. Hence, arterial revascularization of the RCA should only be considered in the presence of severe native vessel stenoses to avoid competitive blood flow, which in turn elevates the risk for early graft occlusion of arterial conduits, such as the RA as well as gastroepiploic artery (GEA) [15,16,17]. Nonetheless, competitive blood flow may also affect venous grafts, accelerating intimal hyperplasia and arteriosclerosis as the main reasons for early occlusion. Higher intraoperatively measured blood flow rates in the single graft group were correlated with longer patency rates, in accordance with the findings of other studies [18,19]. However, coronary vessel diameters did not show any influence on the patency of single conduits in our analysis, keeping in mind that morphological lumen diameters succumb to significant interobserver bias with only marginal difference in our analysis. In a small observational cohort investigation of patients with CTO or subtotal RCA occlusion, Aksut et al. reported that despite narrow lumen diameters of target vessels, anastomosed venous grafts distal from the crux cordis are less prone to occlusion than proximally anastomosed grafts, within a mean follow-up of 67 months [20]. Conversely, we found no correlation between CTO and venous graft patency.

Second, compared to the LCA, which is mainly supplied during diastole, the RCA perfusion occurs in up to 50% of cases during systole [21]. Thinner myocardium and lesser wall stress lead to reduced extravascular compressive forces hindering flow during systole, especially in intramural vessels. This observation contradicts the theory that sequential conduits with higher blood flow may be beneficial compared to single venous conduits. However, if sequential conduits are applied, the target vessel is usually the RPD and not the main RCA proximal from the crux cordis, leading to a limited perfused territory behind the anastomoses. If the RCA is targeted at segments 1–3, the supplied territory becomes significantly larger, especially in the presence of the right dominant type, and even more so if the underlying stenosis is proximal high-grade stenosis or CTO. However, a major influencing factor for sequential graft occlusion may be the runoff within the upstream LCA. Significant stenoses in the LCA system and greater myocardial territories may reduce the runoff capacity of the distal vein graft making the graft prone to competitive native blood flow and revascularization territory of the distal RCA. 

Third, the optimal positioning of the venous conduit is of great importance. Technical challenges are an underestimated factor for the long-term patency of sequential grafts [22]. On the other hand, single bypass grafts also bear the risk of kinking and positioning complications. While these obstacles present risk factors for both single and sequential conduits, they may have more impact on sequential grafts for the revascularization of the right coronary system, as it represents their last run-off region. However, this issue is out of the scope of our study and remains to be studied. 

According to current guidelines, arterial conduits should be chosen as the second and even the third conduit, predominantly based on systematic reviews of RCTs [2,23,24]. However, no unambiguous distinction between the coronary systems has been made. Data from the RAPCO trial (8 years FU) compared specifically the RA (*n* = 112) and SVG (*n* = 304) for grafting of the RCA. No significant differences were found between patency rates and clinical events [25]. The grade of coronary stenosis did not influence the patency of the venous conduits. The RA patency rate was only 64.7% versus 93.3% for the SVG if the grade of RCA stenosis was smaller than 80%. On the other hand, Shi et al. assessed a better risk-adjusted survival in a matched pairs subgroup analysis of 148 patients for grafting the RCA with a RA conduit compared to the SVG (86 ± 6.5% vs. 74 ± 7.8%; *p* = 0.0046) after 15 years [26].

Interestingly, Glineur et al. analyzed data from two randomized controlled trials and found significantly higher patency rates for the SVG compared to the gastroepiploic artery and RA in a protocol-driven 3-year coronary angiography follow-up. The lumen diameter was found to be a relevant predictor for graft patency in arterial conduits to the right coronary system assessing a threshold of under 1.1 mm beneficial for arterial conduit patency. The venous conduits were not affected by lumen diameters [27]. In line with these findings, neither the grade of RCA stenosis nor the diameter of the anastomosed coronary artery did influence graft patency in our study.

Besides age, we found impaired left ventricular function and urgency of CABG procedure both to be relevant risk factors for graft occlusion. Despite outcome improvement for patients with impaired LV function undergoing CABG over medical therapy alone, heart failure remains a relevant risk factor for early graft occlusion and survival [28,29]. Surprisingly, the female gender showed no impact on graft patency in our analysis in contrast to other findings [29]. Larger diameters of the main RCA often addressed in these patients may be responsible for comparable outcomes for both genders since smaller lumen diameter is a contributor to worse outcomes in women after CABG in general.

Interestingly, the presence of diabetes did not correlate with venous graft occlusion to the RCA, like the findings reported by Raza et al. concerning outcome differences in diabetic and non-diabetic patients for long-term patency of the ITA- and the SVG [30]. 

## 5. Conclusions

The revascularization of the RCA will remain controversial, especially regarding the decision between sequential- or single grafting as the technique of choice as graft occlusion has a deeply multifactorial etiology, and our analysis is not representative of the general CABG population. However, with growing evidence leaning towards total arterial revascularization for the left coronary system, single venous bypass grafting to the RCA should retain its value for several reasons: First, in the case of bilateral ITA for complete arterial revascularization, including the RCA is often limited due to graft length. On the other hand, the use of RA as a possible third arterial conduit may not seem feasible if RCA stenosis is not of the highest grade. Moreover, if T/Y-grafted arterial conduits are used for revascularization of multiple vessels, steal phenomena may occur, especially if the radial artery is utilized and anastomosed to the left ITA. 

Thus, targeting the RCA with the SVG will remain part of surgeons’ armamentarium in addition to arterial revascularization of the LCA.

## 6. Strength and Limitations

This is a retrospective observational study and is thus susceptible to the inherent limitations of this type of analysis. It comprises a heterogenic patient cohort without a comparator group, yet it focuses on outcome analyses in a comparatively large group of patients. When the baseline characteristics of patients with single or sequential bypass are compared, significant differences between the cohorts are apparent in several respects. Missing values are consecutively endogenous and are addressed for each variable. In addition, information on the presence of ICD devices was not available in our analysis, and ICD implantation in a CABG population may potentially impact long-term follow-up in multiple aspects [31,32]. Furthermore, information on cardiomyopathies was not included in our analysis. In this context, especially the diagnosis of hypertrophic cardiomyopathy (HCM) is of particular importance as this disease entails thickening of the myocardium and is related to an increased risk of ischemia. Thus, coronary artery disease is found in approximately 20% of HCM patients [33,34]. Moreover, this study mainly included patients who underwent coronary angiography due to recurrent angina and/or acute coronary syndrome during follow-up. Thus, the results may not be representative of all patients who underwent isolated CABG surgery. Several variables and characteristics, such as the specific location of RCA anastomoses and exact graft vessel diameter, were not available for analysis. Moreover, the time to event analysis comprises relevant limitations since the exact time of graft occlusion remains unknown and is vaguely estimated by the time of coronary angiography. This assumption overestimates graft patency in our study. Nevertheless, this report allows for insight into the angiographic follow-up of SVGs directed to the RCA system in symptomatic patients.

## Figures and Tables

**Table 1:** Patient Characteristics at time of CABG.

Patient Characteristics	Total (*n* = 1106)	Sequential Graft (*n* = 289)	Single Graft (*n* = 798)	*p*-Value
Age in years, mean (SD), median (IQR)	63.72 (9.90) 64 (57–71)	66.27 (10.34) 68 (59–74)	62.83 (9.57) 63 (57–70)	<0.001
Female, n (%)	188 (17%)	46 (15.9%)	139 (17.4%)	0.555
BMI, mean (SD), median (IQR)	27.25 (5.75) 27 (25–29) *missing: 8*	27.9 (8.97) 27 (25–29) *missing: 1*	27.01 (4.03) 27 (24–29) *missing: 7*	0.082
euroSCORE (logistic), median (IQR)	6.05 (3.14–10.43) *missing: 332*	7.21 (3.90–11.76) *missing: 33*	5.52 (2.87–9.58) *missing: 294*	0.018
NYHA III-IV, n (%)	830 (75.3%) *missing: 4*	221 (76.5%)	594 (74.4%) *missing: 4*	0.572
Diabetes, n (%)	350 (31.8%) *missing: 7*	106 (36.7%)	240 (30.3%) *missing: 7*	0.054
Smoking, n (%)	513 (51.0%) *missing: 100*	136 (50.2%) *missing: 18*	368 (51.3%) *missing: 80*	0.765
COPD, n (%)	176 (16.1%) *missing: 11*	66 (23.0%) *missing: 2*	107 (13.6%) *missing: 9*	<0.001
Atrial Fibrillation, n (%)	84 (7.6%) *missing: 1*	37 (12.8%) *missing: 1*	46 (5.8%)	0.001
Unstable Angina, n (%)	381 (34.8%) *missing: 10*	91 (31.6%) *missing: 1*	283 (35.9%) *missing: 9*	0.187
Previous MI, n (%)	563 (50.9%)	137 (47.4%)	417 (52.3%)	0.211
Decompensation, n (%)	68 (6.1%)	20 (6.9%)	46 (5.8%)	0.752
Hyperlipidemia, n (%)	894 (82.2%) *missing: 18*	244 (84.4%)	634 (81.3%) *missing: 18*	0.218
Previous cardiothoracic operation, n (%)	39 (3.5%)	8 (2.8%)	31 (3.9%)	0.346
Dialysis/KT/ARF, n (%)	27 (2.4%) *missing: 3*	13 (4.5%)	13 (1.6%) *missing: 3*	0.028
Severely impaired LV-Function, n (%)	104 (10.8%) *missing: 145*	44 (15.2%) *missing: 9*	55 (8.3%) *missing: 136*	0.002
Moderately + severely impaired LV-Function, n (%)	358 (37.3%) *missing: 145*	135 (48.2%) *missing: 9*	212 (32.0%) *missing: 136*	<0.001
Previous PCI, n (%)	388 (35.1%) *missing: 1*	117 (40.5%)	265 (33.2%) *missing: 1*	0.031
Emergency/Urgent indication	426 (38.5%)	94 (32.5%)	328 (41.1%)	0.014

Values are “n (%)—n missing”, “mean ± SD—n missing” or “median (Q1–Q3)—n missing”. Missings are provided if present. BMI, Body-Mass-Index; NYHA, New York Heart Association; COPD, Chronic Obstructive Pulmonary Disease; MI, myocardial Infarction; KT, kidney transplant; ARF, acute renal failure; LV, left ventricular; PCI, percutaneous coronary intervention; SD, standard deviation, IQR, interquartile range.

**Table 2:** RCA, perioperative and follow-up characteristics.

Characteristics	Total (*n* = 1106)	Sequential Graft (*n* = 289)	Single Graft (*n* = 798)	*p*-Value
Total n grafts, mean (SD)	2.81 (0.65)	2.41 (0.66)	2.95 (0.59)	<0.001
CTO, n (%)	174 (20.5%) *missing: 259*	52 (21.8%) *missing: 50*	119 (20.1%) *missing: 206*	0.598
Visible collateralization, n (%)	95 (11.8%) *missing: 301*	28 (12.4%) *missing: 63*	66 (11.7%) *missing: 234*	0.790
Preoperative main RCA stenosis	*missing: 386*	*missing: 90*	*missing: 292*	
0%, n (%)	2 (0.3%)	1 (0.5%)	1 (0.2%)	0.494
25%, n (%)	7 (1.0%)	1 (0.5%)	6 (1.2%)	0.411
50%, n (%)	48 (6.7%)	11 (5.5%)	36 (7.1%)	0.448
75%, n (%)	196 (27.2%)	53 (26.6%)	138 (27.3%)	0.864
90–95%, n (%)	226 (31.4%)	64 (32.2%)	157 (31.0%)	0.771
99%, n (%)	76 (10.6%)	19 (9.5%)	56 (11.1%)	0.557
100%, n (%)	165 (22.9%)	50 (25.1%)	112 (22.1%)	0.396
Graft flow	*missing: 291*	*missing: 104*	*missing: 187*	
Mean graft flow, mL/min ± SD	64.89 ± 35.67	75.87 ± 39.60	61.42 ± 33.78	<0.001
Range graft flow, mL/min	6–340	12–340	6–270	
Total low graft flow < 20 mL/min, n (%)	54 (6.6%)	5 (2.6%)	49 (8.0%)	0.002
**Outcome at follow-up**				
Overall graft occlusion during follow-up, n (%)	368 (33.3%)	101 (34.9%)	260 (32.6%)	0.682
Graft occlusion of CTO, n (%)	69 (39.7%)	20 (38.5%)	47 (39.5%)	0.899
Occlusion of native RCA in follow-up angiogram, n (%)	785 (71%)	171 (59.2%)	604 (75.7%)	<0.001
Patency rates, censored				
1-year, % ± SD	96.5 ± 0.6%	94.2 ± 1.4%	97.1 ± 0.6%	<0.001
5-year, % ± SD	87.4 ± 1.1%	76.9 ± 2.8%	90.4 ± 1.1%	<0.001
10-year, % ± SD	73.4 ± 1.6%	54.8 ± 4.2%	77.8 ± 1.7%	<0.001
median survival in years (SD)		14.39 (1.78)	17.01 (0.58)	<0.001
Intervention native RCA, n (%)	174 (15.8%)	55 (19.0%)	117 (14.7%)	0.125
Intervention bypass graft, n (%)	130 (11.8%)	22 (7.6%)	107 (13.4%)	0.006

Values are “n (%)—n missing”, “mean ± SD—n missing” or “median (Q1–Q3)—n missing”. Missings are provided if present. CTO, chronic total occlusion, RCA, right coronary artery; SD, standard deviation, IQR, interquartile range.

**Table 3:** Univariate/multivariate Cox regression analysis of the complete cohort (outcome variable: graft occlusion (yes/no)).

	Univariate			Multivariate		
Variable	*p*-Value	HR	95% CI	*p*-Value	HR	95% CI
Urgency	0.008	1.281	1.067–1.538	0.003	1.355	1.108–1.656
CTO	0.166	1.213	0.923–1.594			
Visible collateralization	0.123	1.316	0.929–1.864			
Single grafting	<0.001	0.567	0.450–0.715	<0.001	0.575	0.449–0.737
Age	<0.001	1.024	1.013–1.036	0.002	1.019	1.007–1.032
Gender	0.462	1.109	0.842–1.461			
BMI	0.036	1.013	1.001–1.024	0.166	1.008	0.997–1.020
euroSCORE (logistic)	0.159	1.010	0.996–1.024			
Unstable Angina	0.054	0.807	0.649–1.004			
Previous MI	0.850	0.989	0.877–1.114			
NYHA	0.432	1.099	0.869–1.390			
Diabetes	0.128	1.190	0.952–1.487			
Hyperlipidemia	0.445	1.109	0.851–1.444			
Smoking	0.298	1.122	0.903–1.395			
Previous cardiothoracic operation	0.050	0.562	0.316–1.001			
COPD	0.142	1.251	0.928–1.687			
Dialysis/KT/ARF	0.393	1.425	0.633–3.206			
Creatinine	0.478	0.944	0.805–1.107			
Atrial Fibrillation	0.020	1.729	1.091–2.739	0.428	1.216	0.750–1.974
Severely impaired LV-Function,	<0.001	1.931	1.331–2.803	0.001	1.883	1.290–2.748
Previous PTCA	0.678	1.047	0.842–1.302			

CTO, chronic total occlusion; BMI, Body-Mass-Index; NYHA, New York Heart Association; COPD, Chronic Obstructive Pulmonary Disease; MI, myocardial Infarction; KT, kidney transplant; ARF, acute renal failure; LV, left ventricular; PCI, percutaneous coronary intervention.

## Data Availability

Not available.

## Supplementary Materials

The following are available online at https://www.mdpi.com/article/10.3390/jcdd9090285/s1, Table S1: Intraoperative data and graft localization; Table S2: Postoperative data; Table S3: Time to angiography; Table S4: Indication for angiography.

## Abbreviations

CA	coronary artery
CABG	coronary artery bypass grafting
CI	confidence interval
CTO	chronic total occlusion
HCM	hypertrophic cardiomyopathy
HR	hazard ratio
ICU	intensive care unit
ITA	internal thoracic artery
LCA	left coronary artery
RCA	right coronary artery
RMD	ramus marginalis dexter
RPD	right posterior decending
RPLD	ramus posterolateralis dexter

## References

[1] Neumann F.J., Sousa-Uva M., Ahlsson A., Alfonso F., Banning A.P., Benedetto U., Byrne R.A., Collet J.P., Falk V., Head S.J. (2019). 2018 ESC/EACTS Guidelines on myocardial revascularization. Eur. Heart J..

[2] Lawton J.S., Tamis-Holland J.E., Bangalore S., Bates E.R., Beckie T.M., Bischoff J.M., Bittl J.A., Cohen M.G., DiMaio J.M., Don C.W. (2022). 2021 ACC/AHA/SCAI Guideline for Coronary Artery Revascularization: Executive Summary: A Report of the American College of Cardiology/American Heart Association Joint Committee on Clinical Practice Guidelines. Circulation.

[3] Gaudino M., Puskas J.D., Di Franco A., Ohmes L.B., Iannaccone M., Barbero U., Glineur D., Grau J.B., Benedetto U., Taggart D.P. (2017). Three Arterial Grafts Improve Late Survival: A Meta-Analysis of Propensity-Matched Studies. Circulation.

[4] Taggart D.P., Benedetto U., Gerry S., Altman D.G., Gray A.M., Lees B., Gaudino M., Zamvar V., Bochenek A., Buxton B. (2019). Bilateral versus Single Internal-Thoracic-Artery Grafts at 10 Years. N. Engl. J. Med..

[5] Gaudino M., Benedetto U., Fremes S., Ballman K., Biondi-Zoccai G., Nasso G., Raman J., Buxton B., Sedrakyan A., Hayward P.A. (2020). Association of Radial Artery Graft vs Saphenous Vein Graft With Long-term Cardiovascular Outcomes Among Patients Undergoing Coronary Artery Bypass Grafting: A Systematic Review and Meta-analysis. JAMA.

[6] Schwann T.A., Habib R.H., Wallace A., Shahian D.M., O’Brien S., Jacobs J.P., Puskas J.D., Kurlansky P.A., Engoren M.C., Tranbaugh R.F. (2018). Operative Outcomes of Multiple-Arterial Versus Single-Arterial Coronary Bypass Grafting. Ann. Thorac. Surg..

[7] Kim F.Y., Marhefka G., Ruggiero N.J., Adams S., Whellan D.J. (2013). Saphenous vein graft disease: Review of pathophysiology, prevention, and treatment. Cardiol. Rev..

[8] De Vries M.R., Quax P.H.A. (2018). Inflammation in Vein Graft Disease. Front. Cardiovasc. Med..

[9] Caliskan E., de Souza D.R., Böning A., Liakopoulos O.J., Choi Y.-H., Pepper J., Gibson C.M., Perrault L.P., Wolf R.K., Kim K.-B. (2020). Saphenous vein grafts in contemporary coronary artery bypass graft surgery. Nat. Rev. Cardiol..

[10] Goldman S., Zadina K., Moritz T., Ovitt T., Sethi G., Copeland J.G., Thottapurathu L., Krasnicka B., Ellis N., Anderson R.J. (2004). Long-term patency of saphenous vein and left internal mammary artery grafts after coronary artery bypass surgery: Results from a Department of Veterans Affairs Cooperative Study. J. Am. Coll. Cardiol..

[11] Ruttmann E., Dietl M., Feuchtner G.M., Metzler B., Bonaros N., Taggart D.P., Gaudino M., Ulmer H., Benedetto U., Buxton B. (2019). Long-term clinical outcome and graft patency of radial artery and saphenous vein grafts in multiple arterial revascularization. J. Thorac. Cardiovasc. Surg..

[12] FitzGibbon G.M., Leach A.J., Kafka H.P., Keon W.J. (1991). Coronary bypass graft fate: Long-term angiographic study. J. Am. Coll. Cardiol..

[13] Mehta R.H., Ferguson T.B., Lopes R.D., Hafley G.E., Mack M.J., Kouchoukos N.T., Gibson C.M., Harrington R.A., Califf R.M., Alexander J.H. (2011). Saphenous vein grafts with multiple versus single distal targets in patients undergoing coronary artery bypass surgery: One-year graft failure and five-year outcomes from the Project of Ex-Vivo Vein Graft Engineering via Transfection (PREVENT) IV trial. Circulation.

[14] Li J., Liu Y., Zheng J., Bai T., Liu Y., Wang X., Liu N., Cheng L., Chen Y., Zhang H. (2011). The Patency of Sequential and Individual Vein Coronary Bypass Grafts: A Systematic Review. Ann. Thorac. Surg..

[15] Pinho-Gomes A.-C., Azevedo L., Antoniades C., Taggart D.P. (2018). Comparison of graft patency following coronary artery bypass grafting in the left versus the right coronary artery systems: A systematic review and meta-analysis. Eur. J. Cardio-Thorac. Surg..

[16] Sabik J.F., Lytle B.W., Blackstone E.H., Khan M., Houghtaling P.L., Cosgrove D.M. (2003). Does competitive flow reduce internal thoracic artery graft patency?. Ann. Thorac. Surg..

[17] Sabik J.F., Blackstone E.H., Houghtaling P.L., Cosgrove D.M. (2005). Comparison of saphenous vein and internal thoracic artery graft patency by coronary system. Ann. Thorac. Surg..

[18] Zhao Z., Fu C., Zhang L.-X., Zhang G.-D., Chen Y. (2020). Perioperative observations of different bypass modes of a right coronary system based on instantaneous blood flow during the operation. J. Cardiothorac. Surg..

[19] Nordgaard H., Vitale N., Haaverstad R. (2009). Transit-Time Blood Flow Measurements in Sequential Saphenous Coronary Artery Bypass Grafts. Ann. Thorac. Surg..

[20] Aksut M., Koksal C., Kocamaz O., Aksoy E., Kara I., Onk A., Ozkaynak B. (2012). Should right coronary bypass grafts be anastomosed proximal or distal to the crux? A comparison of graft patencies. Ann. Thorac. Cardiovasc. Surg..

[21] Marcus M., Wright C., Doty D., Eastham C., Laughlin D., Krumm P., Fastenow C., Brody M. (1981). Measurements of coronary velocity and reactive hyperemia in the coronary circulation of humans. Circ. Res..

[22] Moshkovitz Y., Raanani E. (2016). The art of saphenous vein grafting and patency maintenance. J. Thorac. Cardiovasc. Surg..

[23] Gaudino M., Benedetto U., Fremes S., Biondi-Zoccai G., Sedrakyan A., Puskas J.D., Angelini G.D., Buxton B., Frati G., Hare D.L. (2018). Radial-Artery or Saphenous-Vein Grafts in Coronary-Artery Bypass Surgery. N. Engl. J. Med..

[24] Cao C., Ang S.C., Wolak K., Peeceeyen S., Bannon P., Yan T.D. (2013). A meta-analysis of randomized controlled trials on mid-term angiographic outcomes for radial artery versus saphenous vein in coronary artery bypass graft surgery. Ann. Cardiothorac. Surg..

[25] Hadinata I.E., Hayward P.A., Hare D., Matalanis G.S., Seevanayagam S., Rosalion A., Buxton B.F. (2009). Choice of Conduit for the Right Coronary System: 8-Year Analysis of Radial Artery Patency and Clinical Outcomes Trial. Ann. Thorac. Surg..

[26] Shi W.Y., Hayward P.A., Fuller J.A., Tatoulis J., Rosalion A., Newcomb A.E., Buxton B.F. (2016). Is the radial artery associated with improved survival in older patients undergoing coronary artery bypass grafting? An analysis of a multicentre experiencedagger. Eur. J. Cardiothorac. Surg..

[27] Glineur D., D’Hoore W., de Kerchove L., Noirhomme P., Price J., Hanet C., El Khoury G. (2011). Angiographic predictors of 3-year patency of bypass grafts implanted on the right coronary artery system: A prospective randomized comparison of gastroepiploic artery, saphenous vein, and right internal thoracic artery grafts. J. Thorac. Cardiovasc. Surg..

[28] Velazquez E.J., Lee K.L., Jones R.H., Al-Khalidi H.R., Hill J.A., Panza J.A., Michler R.E., Bonow R.O., Doenst T., Petrie M.C. (2016). Coronary-Artery Bypass Surgery in Patients with Ischemic Cardiomyopathy. N. Engl. J. Med..

[29] Arif R., Farag M., Gertner V., Szabó G., Weymann A., Veres G., Ruhparwar A., Bekeredjian R., Bruckner T., Karck M. (2016). Female Gender and Differences in Outcome after Isolated Coronary Artery Bypass Graft Surgery: Does Age Play a Role?. PLoS ONE.

[30] Raza S., Blackstone E.H., Houghtaling P.L., Rajeswaran J., Riaz H., Bakaeen F.G., Lincoff A.M., Sabik J.F. (2017). Influence of Diabetes on Long-Term Coronary Artery Bypass Graft Patency. J. Am. Coll. Cardiol..

[31] Fumagalli S., Pieragnoli P., Haugaa K.H., Potpara T.S., Rasero L., Ramacciati N., Ricciardi G., Solimene F., Mascia G., Mascioli G. (2019). The influence of age on the psychological profile of patients with cardiac implantable electronic devices: Results from the Italian population in a multicenter study conducted by the European Heart Rhythm Association. Aging.

[32] Al-Dadah A.S., Voeller R.K., Rahgozar P., Lawton J.S., Moon M.R., Pasque M.K., Damiano R.J., Moazami N. (2007). Implantable cardioverter-defibrillators improve survival after coronary artery bypass grafting in patients with severely impaired left ventricular function. J. Cardiothorac. Surg..

[33] Wang S., Cui H., Tang B., Zhu C., Meng L., Yu Q., Huang X., Wu R., Wang S. (2019). Mid-term outcomes of simultaneous coronary artery bypass graft surgery and septal myectomy in patients with hypertrophic obstructive cardiomyopathy: A case-controlled study. J. Card. Surg..

[34] Mascia G., Crotti L., Groppelli A., Canepa M., Merlo A.C., Benenati S., Di Donna P., Della Bona R., Soranna D., Zambon A. (2022). Syncope in hypertrophic cardiomyopathy (part I): An updated systematic review and meta-analysis. Int. J. Cardiol..

