# Supplementary material for: Long-Term Patency of Venous Conduits Targeting the Right Coronary Artery System—Single Is Superior to Sequential bypass Grafting
Source: J Cardiovasc Dev Dis. 2022 Aug 26;9(9):285. doi: 10.3390/jcdd9090285 (PMC9506273; doi:10.3390/jcdd9090285)
Supplement: Supplementary file 1 [file jcdd-09-00285-s001.zip › jcdd-1866677-supplementary.pdf]

Supplement Table S1. Intraoperative data and graft localization

Intraoperative characteristics	Total (n=1106)	Sequential graft (n=289)	Single graft (n=798)
EC, n (%)	1095 (99%)	288 (99.7%)	789 (98.9%)
Operation time in min, mean (SD), median (IQR)	226.60 (67.43), 220 (180 – 265)	233.76 (72.69), 225 (190 – 270)	224.05 (65.80), 210 (179 – 265)
CBP time in min, mean (SD), median (IQR)	107.70 (38.34), 102 (82 – 127) *missing: 6*	110.57 (44.29), 105 (82 – 131.75) *missing: 1*	106.47 (36.17), 101 (81 -126) *missing: 4*
Cross-clamp time in min, mean (SD), median (IQR)	60.69 (22.03), 58 (47 – 73) *missing: 6*	63.33 (23.83), 62 (49 – 75) *missing: 1*	59.73 (21.32), 57 (46 – 72) *missing: 5*
Blood transfusion in ml, mean (SD), median (IQR)	606.10 (655.86), 600 (0 – 900)	644.29 (664.60), 600 (0 – 900)	594.86 (649.02), 600 (0 – 900)
FFP in ml, mean (SD), median (IQR)	188.79 (357.92), 0 (0 – 400)	135.81 (335.73), 0 (0 – 0)	206.83 (361.09), 0 (0 – 400)
IABP, n (%)	38 (3.4%)	7 (2.4%)	31 (3.9%)
Min. temperature in °C, mean (SD), median (IQR)	32.51 (2.44), 32.7 (31.5 – 34) *missing: 7*	33.56 (1.82), 34.0 (32.8 – 34.3) *missing: 1*	32.12 (2.54), 32.3 (31.1 – 33.7) *missing: 5*
**Graft localization**			
RCA, n (%)	627 (51.6%)	55 (16.7%)	565 (65.5%)
Diameter in mm, mean (SD, Range)	1.607 (0.302, 0.8 – 4.0) *missing: 4*	1.507 (0.267, 1.0 – 2.5)	1.616 (0.304, 0.8 – 4.0) *missing: 4*
RPD, n (%)	409 (33.6%)	212 (64.2%)	187 (21.7%)
Diameter in mm, mean (SD, Range)	1.463 (0.251, 0.5 – 3.0) *missing: 1*	1.456 (0.248, 0.5 – 3.0) *missing: 1*	1.473 (0.253, 0.9 – 3.0)
RMD, n (%)	178 (14.6%)	61 (18.5%)	110 (12.8%)
Diameter in mm, mean (SD, Range)	1.433 (0.243, 1.0 – 2.5)	1.433 (0.245, 1.0 – 2.0)	1.426 (0.225, 1.0 – 2.5)
RPLD, n (%)	2 (0.2%)	2 (0.6%)	0 (0%)
Diameter in mm, mean (SD, Range)	1.250 (0.354, 1.0 – 1.5)	1.250 (0.354, 1.0 – 1.5)	
**Graft flow**			
Mean graft flow ml/min (SD)	64.89 (35.67) *missing: 291*	75.87 (39.60) *missing: 104*	61.42 (33.78) *missing: 187*
Graft flow <20 ml/min, n (%)	54 (6.6%)	5 (2.6%)	49 (8.0%)
**Graft Quality**			
Sufficient/good: 985<br>Small diameter: 54<br>Large diameter: 39<br>Sclerosis: 7<br>Varicosis: 8<br>*missing: 13*			

Values are “n (%) – n missing”, “mean ± SD – n missing” or “median (Q1-Q3) – n missing”. Missings are provided if present. EC, extracorporeal circulation; CBP, cardiopulmonary bypass, FFP, fresh frozen plasma; IABP, intraaortic ballon pump; RCA, right coronary artery; RPD, right posterior descending; RMD, ramus marginalis dexter; RPLD, ramus posterolateralis dexter; SD, standard deviation, IQR, interquartile range

Supplement Table S2. Postoperative data

**Outcome characteristics**	**Total (n=1106)**	**Sequential graft (n=289)**	**Single graft (n=798)**
Low output, n (%)	32 (3.8%) *missing: 260*	6 (2.2%) *missing: 22*	26 (4.6%) *missing: 236*
Postoperative atrial fibrillation, n (%)	101 (11.6%) *missing: 236*	38 (13.8%) *missing: 13*	60 (10.4%) *missing: 222*
Re-bleeding, n (%)	67 (7.7%) *missing: 233*	23 (8.3%) *missing: 13*	44 (7.6%) *missing: 219*
CV accident, n (%)	2 (0.2%) *missing: 233*	1 (0.4%) *missing: 13*	1 (0.2%) *missing: 219*
Cardiac arrest / CPR, n (%)	13 (1.5%) *missing: 233*	2 (0.7%) *missing: 13*	11 (1.9%) *missing: 219*
Re-thoracotomy	29 (3.2%) *missing: 233*	11 (4.0%) *missing: 13*	18 (3.1%) *missing: 219*

Values are “n (%) – n missing”, “mean ± SD – n missing” or “median (Q1-Q3) – n missing”. Missings are provided if present. CV, cerebrovascular; CPR, cardiopulmonary resuscitation

Supplement Table S3. Time to angiography

**Interval to angiography**	**Number of patients**
1 year	71
5 years	306
10 years	878
>10 years	228

Supplement Table S4. Indication for angiography

**Indication for angiography**	**Number of patients**
Elective (before intervention TAVR, PMVR,…)	48
Urgent (suspected progress of CAD)	632
Emergent (STEMI, NSTEMI, uAP)	426

TAVR, transcatheter aortic valve replacement; PMVR, percutaneous mitral valve repair; CAD, coronary artery disease; STEMI, ST-elevation myocardial infarction; NSTEMI, non-ST-elevation myocardial infarction; uAP, unstable angina
